# Cholestasis in preterm infants: when is a yellow alert?

**DOI:** 10.1186/1824-7288-40-S2-A12

**Published:** 2014-10-09

**Authors:** Silvia Nastasio, Giuseppe Maggiore

**Affiliations:** 1 Department of Clinical and Experimental Medicine, Pediatric Gastroenterology and Hepatology, University Hospital of Pisa, 56127 Pisa, Italy

## 

Besides the transient bilirubin transport immaturity, preterm infants are particularly at risk for different forms and degrees of bile formation impairment because of metabolic demands that are not matched by functional maturation in the first weeks of life.

Cholestasis, affecting approximately 1 of 2 500 infants, is more commonly reported in preterm infants with an incidence varying between 10 and 20%, and it is mainly due to a combination of factors including delayed enteral nutrition, low birth weight, prolonged parenteral nutrition, hypoxia, infection, liver ischemia, immaturity of bile acid metabolism, surgical procedures and multiple drug treatments [1]. This reality of the setting is defined as *transient or multifactorial* cholestasis [2], which is the most frequent form of cholestasis in neonatal intensive care unit, usually transient and followed by a gradual full recovery [3].

Cholestasis in preterm infants may however also be indicative of a severe liver disease such as biliary atresia (BA) or other biliary tract disorders. The development of a persistent cholestatic jaundice, even in presence of colored stools, incentives to conduct a thorough investigation [4]. Abdominal ultrasonography may support the diagnosis of BA showing: absence of gallbladder, the “triangular cord” sign or a cyst located at porta hepatis. Transient multifactorial cholestasis is a diagnosis of exclusion and a definitive diagnosis can be made only after the complete resolution of the clinical picture. Moreover, diagnosis of biliary atresia in preterm jaundiced neonates is difficult since discoloration of stools can occur several weeks after birth [4].

Besides specific cholestatic disorders for which specific medical and surgical treatment are available, there is no unequivocal evidence that any medical treatment alters the natural history of multifactorial cholestasis. Treatment with ursodeoxycholic acid may indeed be useful, although there is no evidence of effectiveness. Every effort must be made to remove all risk factors for liver injury. Consequences of cholestasis also need to be managed by administering fat -soluble vitamins, especially vitamin K and nutritional formulas containing medium-chain fatty acids.

Although multifactorial transient cholestasis is the most common cause of prolonged jaundice in preterm infants, neonatologists need to be aware that premature infants can also present with signs of severe liver disease. To avoid any diagnostic delay it is mandatory to promptly identify all the conditions suitable for an early and specific treatment with a structured approach to the investigation of cholestasis tailored to the preterm infant [5].
